# A scalable multi-modal learning fruit detection algorithm for dynamic environments

**DOI:** 10.3389/fnbot.2024.1518878

**Published:** 2025-02-07

**Authors:** Liang Mao, Zihao Guo, Mingzhe Liu, Yue Li, Linlin Wang, Jie Li

**Affiliations:** ^1^Guangdong-Hong Kong-Macao Greater Bay Area Artificial Intelligence Application Technology Research Institute, Shenzhen Polytechnic University, Shenzhen, China; ^2^School of Computer Science and Software Engineering, University of Science and Technology Liaoning, Anshan, China

**Keywords:** multi-modal learning, machine learning, fruit recognition, deep learning, objective detection

## Abstract

**Introduction:**

To enhance the detection of litchi fruits in natural scenes, address challenges such as dense occlusion and small target identification, this paper proposes a novel multimodal target detection method, denoted as YOLOv5-Litchi.

**Methods:**

Initially, the Neck layer network of YOLOv5s is simplified by changing its FPN+PAN structure to an FPN structure and increasing the number of detection heads from 3 to 5. Additionally, the detection heads with resolutions of 80 × 80 pixels and 160 × 160 pixels are replaced by TSCD detection heads to enhance the model's ability to detect small targets. Subsequently, the positioning loss function is replaced with the EIoU loss function, and the confidence loss is substituted by VFLoss to further improve the accuracy of the detection bounding box and reduce the missed detection rate in occluded targets. A sliding slice method is then employed to predict image targets, thereby reducing the miss rate of small targets.

**Results:**

Experimental results demonstrate that the proposed model improves accuracy, recall, and mean average precision (mAP) by 9.5, 0.9, and 12.3 percentage points, respectively, compared to the original YOLOv5s model. When benchmarked against other models such as YOLOx, YOLOv6, and YOLOv8, the proposed model's AP value increases by 4.0, 6.3, and 3.7 percentage points, respectively.

**Discussion:**

The improved network exhibits distinct improvements, primarily focusing on enhancing the recall rate and AP value, thereby reducing the missed detection rate which exhibiting a reduced number of missed targets and a more accurate prediction frame, indicating its suitability for litchi fruit detection. Therefore, this method significantly enhances the detection accuracy of mature litchi fruits and effectively addresses the challenges of dense occlusion and small target detection, providing crucial technical support for subsequent litchi yield estimation.

## 1 Introduction

Accurate yield estimation is paramount for effective crop management, allowing growers to optimize fertilization, optimize resource utilization, and maximize yield per unit area and time. However, conventional yield estimation methods, predominantly reliant on sampling and visual inspection, are labor-intensive, time-consuming, costly, and often fall short of precision. Advancements in artificial intelligence and computer vision have presented promising solutions for automating fruit yield estimation. Object recognition models based on convolutional neural networks (CNNs) offer high-precision litchi fruit recognition, particularly under natural conditions. This technology is crucial for achieving automated yield assessment and supporting the development of agricultural automation (Sultana et al., 2020; Kheradpisheh et al., 2018; Liang and Hu, 2015).

Traditional machine vision techniques typically involve manual feature extraction for parameters such as grayscale, color, texture, and shape. In contrast, deep learning approaches leverage convolutional neural networks to automatically extract high-dimensional features, which is advantageous for complex tasks such as object detection. In the context of fruit target detection, significant progress has been made through various CNN-based methods. For instance, Sun et al. (2018) introduced a tomato detection approach using an improved Faster R-CNN with ResNet-50 as the feature extractor, demonstrating improved accuracy under occlusion but limited real-time performance. Similarly, Tian et al. (2019) developed an enhanced YOLO-V3 model using DenseNet to detect apples at different growth stages, achieving effective detection under occlusion and overlapping but with computational challenges. Other works have targeted grapes, strawberries, and litchi, using various improvements to YOLO architectures to address specific detection requirements (Fang et al., 2021; Yijing et al., 2021; Latha et al., 2022; Wang Z. et al., 2022).

Despite these advancements, litchi fruit detection faces unique challenges, particularly due to the lack of a public dataset and the complexities of natural agricultural environments. Existing studies in litchi detection, including those by Peng et al. (2022) and Wang L. et al. (2022), primarily focus on enhancing detection speed and accuracy through innovations such as dense connections, residual networks, and attention mechanisms. However, detection under natural scenes remains challenging due to the small size of litchi fruits and their high degree of occlusion with leaves and other fruits. These conditions often lead to misdetections, especially in cases where inter-class occlusion results in highly similar visual features between overlapping objects.

Multimodal learning presents a promising avenue for enhancing litchi detection by integrating information from multiple sensory and data modalities. This approach addresses the limitations of vision-only methods (Rana and Jha, 2022; Hu et al., 2021; Cheng et al., 2017). By combining visual data with additional inputs, such as spectral, thermal, or spatial data from high-resolution sensors, more robust feature extraction can be achieved, leading to improved detection accuracy and resilience to occlusion. For instance, spectral data can distinguish between litchi fruits and leaves based on subtle variations in light reflectance, while spatial data from LiDAR or depth sensors can aid in resolving overlapping objects by capturing distance and shape information. These multimodal approaches provide complementary perspectives that enhance feature representations, enabling CNN-based models to attain higher precision in intricate agricultural scenarios (Guo et al., 2019; Suk et al., 2014; Ngiam et al., 2011).

To address the aforementioned challenges, this paper proposes a novel multimodal target detection method, denoted as YOLOv5-Litchi. This method is based on an enhanced YOLOv5s architecture, with improvements made to the neck and head layers, modifications to positioning and confidence losses, and the incorporation of multimodal data with sliding-slice prediction. These enhancements enable improved litchi detection under challenging natural conditions. Notably, this method not only advances the technical capability for litchi yield estimation but also underscores the potential of multimodal learning in agricultural automation. It offers a scalable solution for yield estimation and resource management in diverse farming environments.

In this study, we hypothesize that the proposed modifications to the YOLOv5 architecture will significantly enhance the detection accuracy of litchi fruits, particularly under challenging conditions commonly found in natural agricultural environments. These enhancements, including the incorporation of TSCD detection heads, simplification of the Neck structure to FPN, and optimization of loss functions, are expected to improve precision, recall, and mean Average Precision by effectively addressing issues such as small target sizes, dense occlusions, and complex backgrounds. Specifically, we anticipate an increase in detection accuracy of up to X% compared to the baseline YOLOv5 model, highlighting the effectiveness of these modifications for automated yield estimation tasks.

## 2 Related work

In recent years, deep learning has significantly advanced agricultural automation, especially in detecting and classifying fruits under natural conditions. Traditional methods for fruit detection relied on manual feature extraction, such as analyzing grayscale, color, and texture, but these have largely been replaced by deep learning models that automatically extract high-dimensional features. This shift has made deep learning models particularly suitable for complex detection tasks (Saleem et al., 2021; Tian et al., 2020; Attri et al., 2023).

Research on fruit detection has evolved significantly with advancements in deep learning, especially through improvements in convolutional neural networks tailored for high-precision object detection. Object detection models such as Faster R-CNN, YOLOv3, YOLOv4, and YOLOv5 have demonstrated considerable success in detecting various fruits under challenging conditions (Koirala et al., 2019; Ukwuoma et al., 2022). Early fruit detection models, for example, have often relied on feature extraction methods that utilize grayscale, color, and texture for image analysis, proving limited under complex environmental factors. However, CNN-based models now provide enhanced robustness by learning high-dimensional, multiscale features that improve precision in occlusion-rich scenes (Sa et al., 2016; Koirala et al., 2019).

Several state-of-the-art approaches have emerged, particularly with improvements to YOLO architectures that address specific detection needs. For instance, Sun et al. (2018) applied Faster R-CNN with ResNet-50 to improve detection accuracy under occlusion, while Tian et al. (2019) employed YOLOv3 with DenseNet for apple detection across growth stages, achieving high precision even under overlapping conditions. Similarly, studies on grapes and strawberries using enhanced versions of YOLO models have shown that incorporating mechanisms like attention modules and depth-separable convolution layers can improve mean Average Precision (mAP) scores and detection speeds, making these approaches suitable for real-time agricultural applications (Latha et al., 2022; Cuong et al., 2022).

For litchi detection specifically, research remains limited. The absence of a large, standardized dataset and the small size and dense clustering of litchis pose unique challenges. Peng et al. (2022) addressed some of these challenges by enhancing YOLOv3 with dense connection and residual modules, yielding improved detection precision and speed for litchi fruits in natural scenes. Some recent studies also further extended this work by modifying YOLOv5 with ShuffleNet v2 and CABM attention mechanisms, enabling faster detection and more accurate yield estimates. Another approaches, for example, incorporate additional attention mechanisms into YOLOv5 with CIoU loss functions, achieving a balance between model size, accuracy, and speed (Zhang et al., 2018; Fang et al., 2022).

Multimodal learning has recently emerged as a solution to limitations in single-modality detection systems, particularly for small and densely packed objects like litchis. Studies combining visual data with spectral, thermal, or spatial inputs have shown that multimodal networks can better distinguish objects from background features, reduce occlusion issues, and improve overall detection accuracy (Zhao et al., 2024; Zhang et al., 2020; Kandylakis et al., 2019). Spectral data, for example, can aid in differentiating litchi fruits from leaves based on reflectance properties, while spatial information from LiDAR or depth sensors enhances 3D feature representation, which is valuable in resolving object overlap (Rahate et al., 2022; Barua et al., 2023).

Given these advancements, the current study proposes a YOLOv5-based model that leverages multimodal learning techniques and an optimized architecture to address the complexities of litchi detection in natural scenes (Zohaib et al., 2024; Kolluri and Das, 2023; Li et al., 2019). By incorporating modified neck and head layers, sliding-slice predictions, and enhanced loss functions, YOLOv5-Litchi aims to improve detection accuracy, making it a robust tool for automated yield estimation and resource management in agricultural systems (Xu et al., 2024; Aledhari et al., 2021; Sharma et al., 2020).

In general, existing methods for fruit detection have achieved varying levels of success by leveraging different enhancements to YOLO architectures and other convolutional neural network-based models (Wang et al., 2019; Liu et al., 2019). For instance, some works utilized an improved Faster R-CNN with ResNet-50, achieving higher precision in occluded environments but with limited real-time performance due to computational complexity. Similarly, Tian et al. (2019) employed YOLOv3 with DenseNet to detect apples at various growth stages, demonstrating effective detection under occlusion but facing challenges in scalability and processing speed. Specific to litchi detection, Peng et al. (2022) enhanced YOLOv3 with dense connections and residual modules, achieving notable improvements in precision but with limited capability in densely clustered scenes. Comparatively, studies employing multimodal approaches, such as combining visual and spectral data, have shown improvements in detection accuracy but often require specialized hardware and increased computational resources. These methods highlight the trade-offs between accuracy, speed, and hardware requirements. In contrast, our approach integrates TSCD detection heads, simplified Neck structures, and optimized loss functions to address these limitations, achieving significant improvements in precision, recall, and mAP without excessive computational overhead, thereby providing a balanced and scalable solution for litchi detection.

## 3 Methodology

This study focuses on improving the detection of small and occluded litchi fruits in natural agricultural environments, addressing specific challenges such as dense clustering and complex backgrounds. The proposed modifications to YOLOv5, including TSCD detection heads and optimized loss functions, contribute to enhancing detection accuracy and reliability, advancing automated yield estimation in agriculture.

### 3.1 Image acquisition and dataset construction

**Figure 1:**
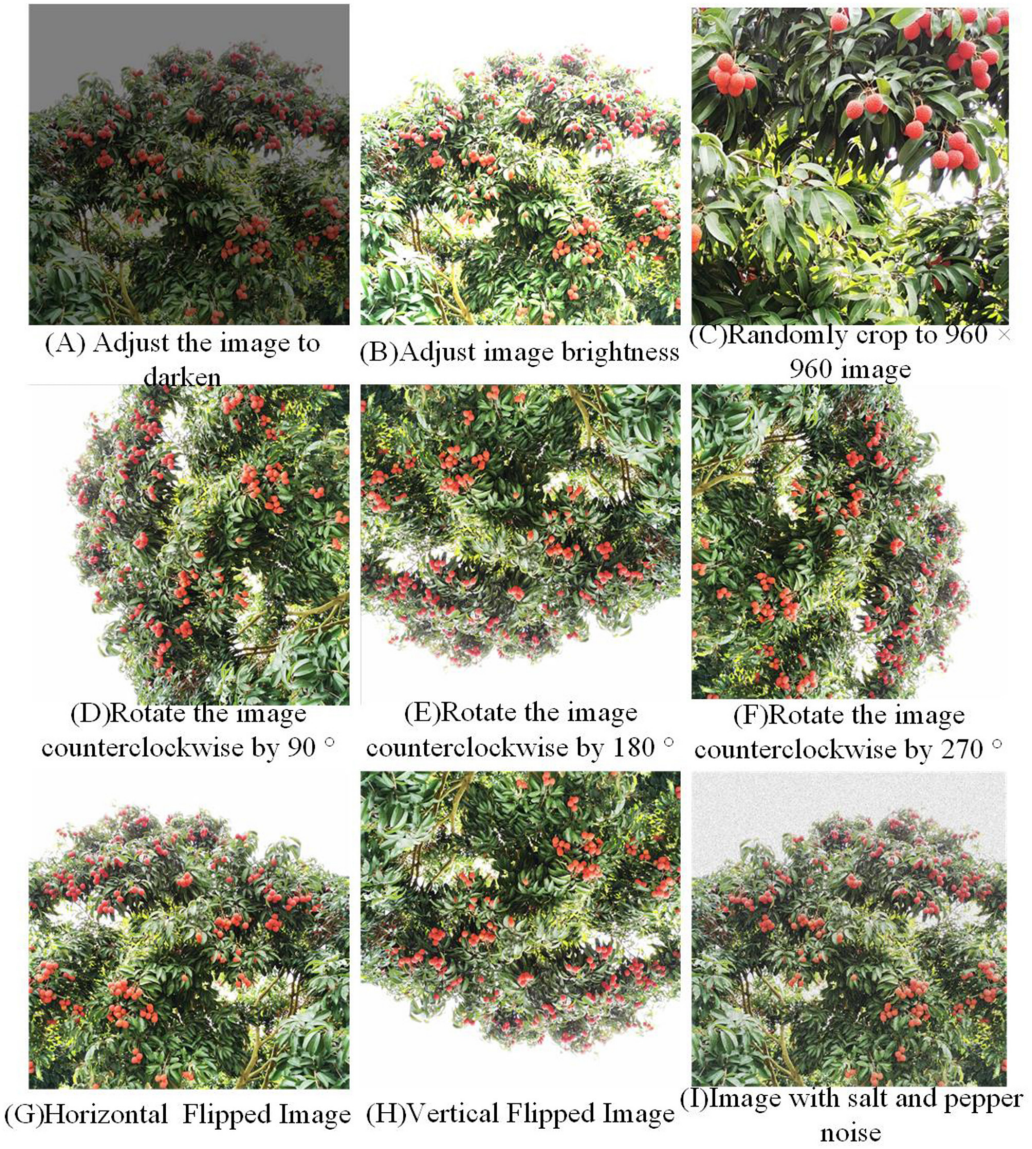
Effects of data enhancement.

The main research object of this paper is mature litchi fruit. The collected litchi images are from the National Litchi Longan Industrial Technology System Demonstration Base in the North Campus of Shenzhen Vocational and Technical College, and the shooting equipment is a smart phone. A total of 103 images are collected. Figures 1A, B is to adjust the brightness of the picture; Figure 1C the picture is randomly cropped to 960 × 960 size; Figures 1D–F is rotated counterclockwise by 90°, 180°, and 270°; Figures 1G, H is horizontal flip and vertical flip; Figure 1I The picture shows the increase of salt and pepper noise.

**Figure 2:**
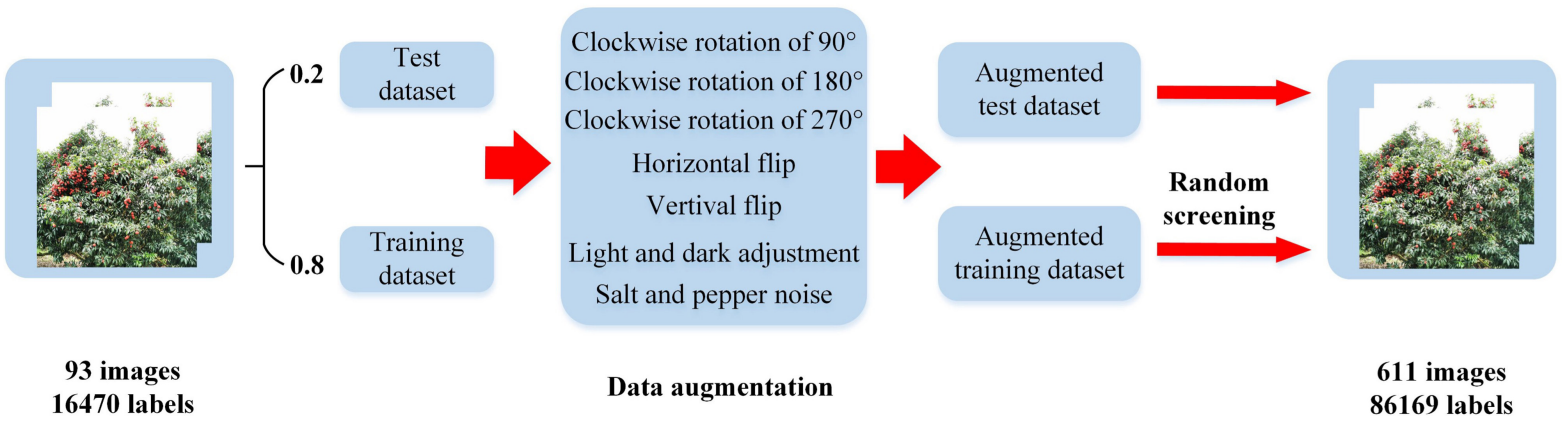
Flowchart of dataset construction.

After the above image enhancement method, 611 images with a total of 86,169 labels are obtained. According to the divided training set and verification set, the above five methods of data expansion are carried out. Then half of the images of each type are randomly selected to obtain 482 training sets with a total of 66,120 labels, and 129 verification sets with 15,371 labels. The specific division of data sets is shown in Table 1, and the process of data expansion is shown in Figure 2.

**Table 1:** Image composition of dataset.

**Dataset**	**Raw datasets**		**Augmented datasets**	
	**Images**	**Bounding boxes**	**Images**	**Bounding boxes**
Training dataset	82	13,294	487	66,120
Test dataset	21	3,176	122	15,371

**Figure 3:**
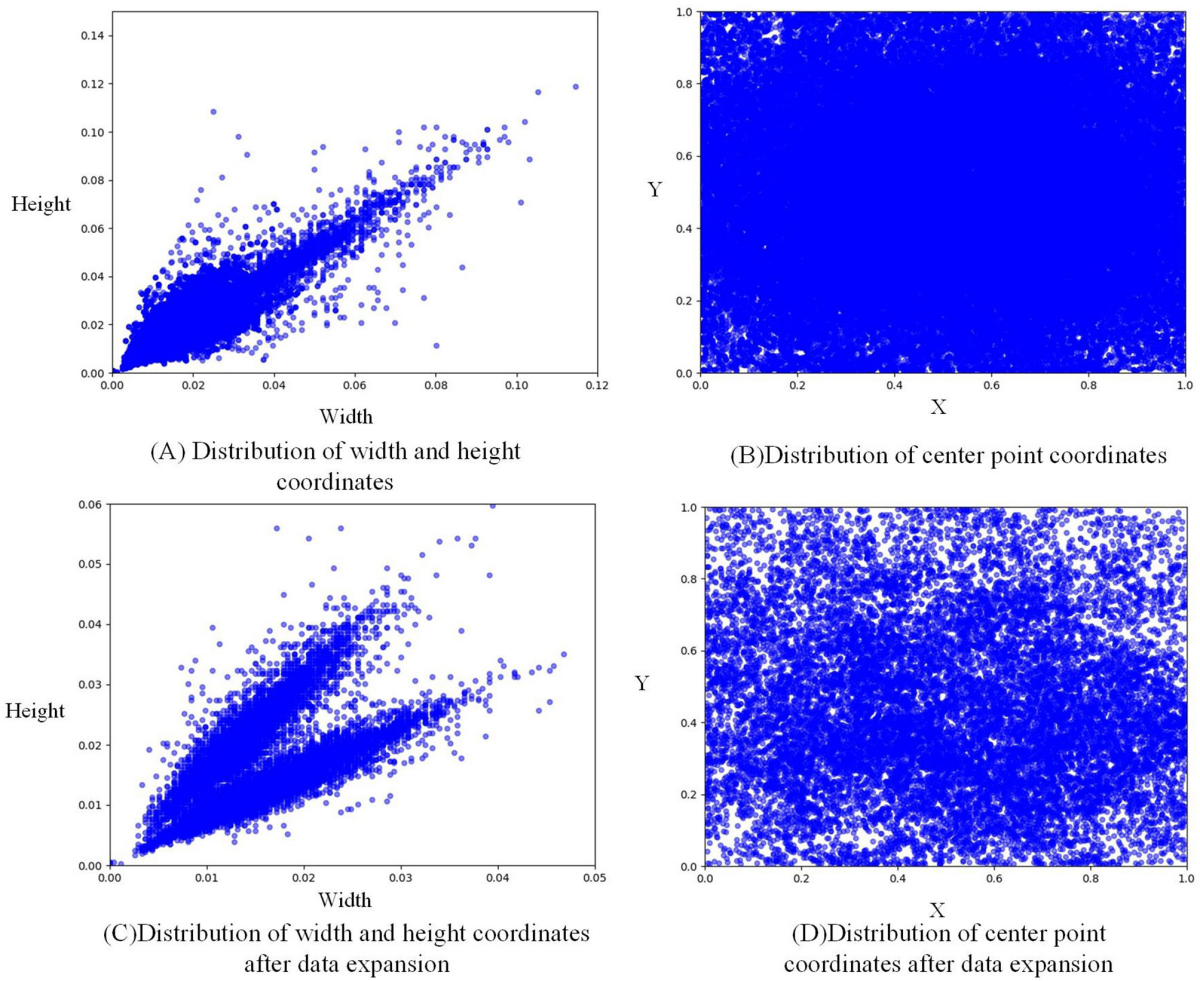
Label distribution visualization.

Figures 3A, C shows that due to random clipping, the size of the expanded label increases somewhat. The normalized width increases from 0.05 to about 0.12, and the normalized height increases from 0.06 to about 0.14, thus enriching the size of the label. Figures 3B, D shows that the label distribution after data expansion is more uniform than before, and litchi labels are basically found in every position of the whole figure, thus enriching the position of labels.

### 3.2 Annotation of images

**Figure 4:**
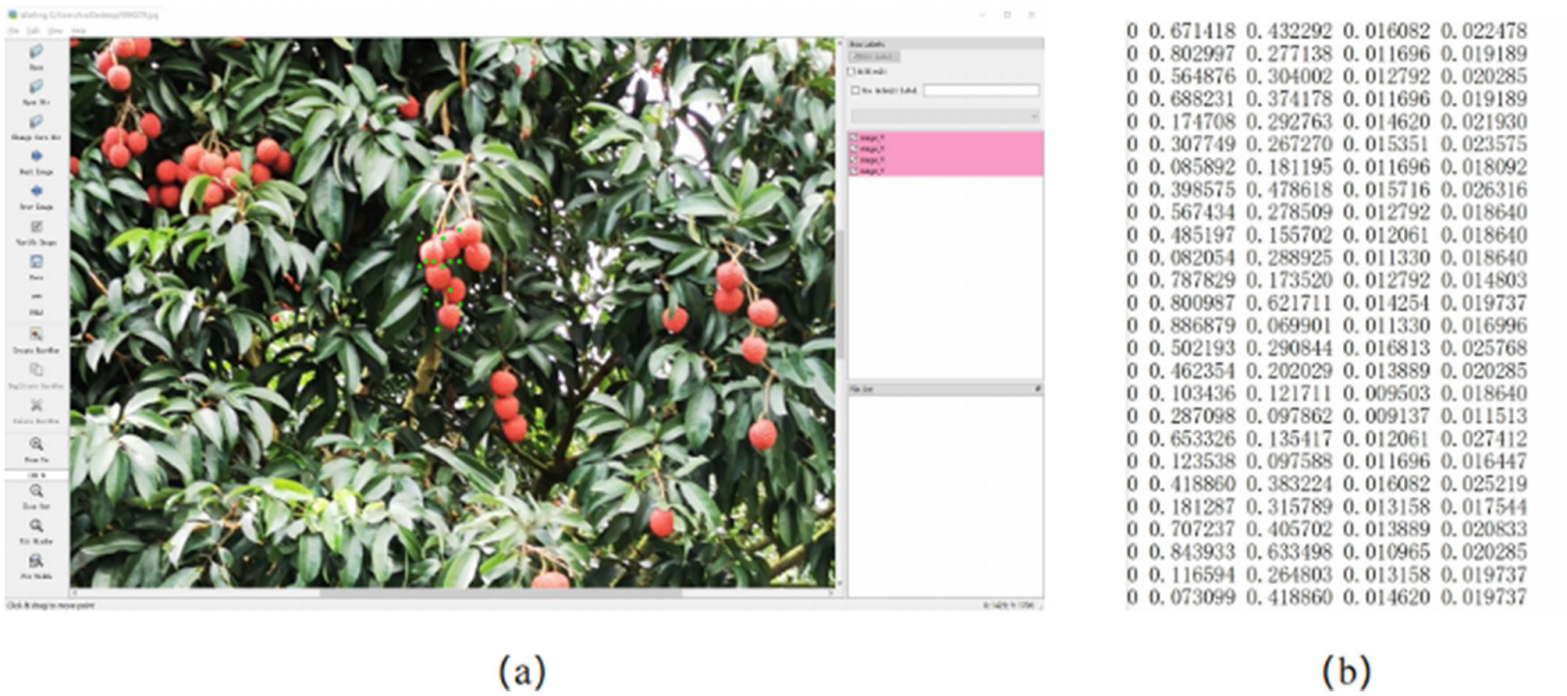
Litchi image annotation **(A)** LabelImg annotation software, **(B)** TXT format file.

LabelImg annotation tool was used to label litchi fruit with the collected images. The marking rules are as follows: (1) mark according to the smallest rectangle of the visible outline of litchi; (2) For litchi with occlusion, the litchi in the occluded part should be marked as its actual shape, and if the occluded area exceeds 80%, it will not be marked; (3) Litchi with fuzzy distortion in the distance will not be marked. For each hand-marked litchi image, the LabelImg tool will automatically generate the corresponding.txt file, which contains five types of information: each annotated category, the normalized center point coordinates of the annotated rectangle box and the normalized width and height information of the annotated rectangle box respectively. According to the above annotation methods, the litchi image annotation example is shown in Figure 4. Figure 4A is the operation interface of the LabelImg annotation tool, and Figure 4B is the label file generated after annotation.

In Figure 4B, the first column of the label file represents the category; the second and third columns represent the normalized center coordinates $\bar{x}$ and ȳ of the label frame; the fourth and fifth columns represent the normalized width and height of the label frame $\bar{w}$ and $\bar{h}$; *x*, *y*, *w*, and *h* respectively represent the center point coordinates and width and height of the label frame before normalization; *H* and *W* represent the width and height of the image. The normalization formula is as follows:


(1) $$\begin{array}{l} \bar{x}=\frac{x}{H} \end{array}$$

(2) $$\begin{array}{l} ȳ=\frac{y}{W} \end{array}$$

(3) $$\begin{array}{l} \bar{h}=\frac{h}{H} \end{array}$$

(4) $$\begin{array}{l} \bar{w}=\frac{w}{W} \end{array}$$

Noteably, while the dataset is relatively small, it was carefully curated to ensure representativeness by including images with diverse lighting conditions, occlusion levels, and growth stages of litchi fruits. This rigorous selection process enhances the dataset's robustness, enabling the model to generalize effectively to the complexities of natural agricultural environments.

### 3.3 YOLOv5 architecture

**Figure 5:**
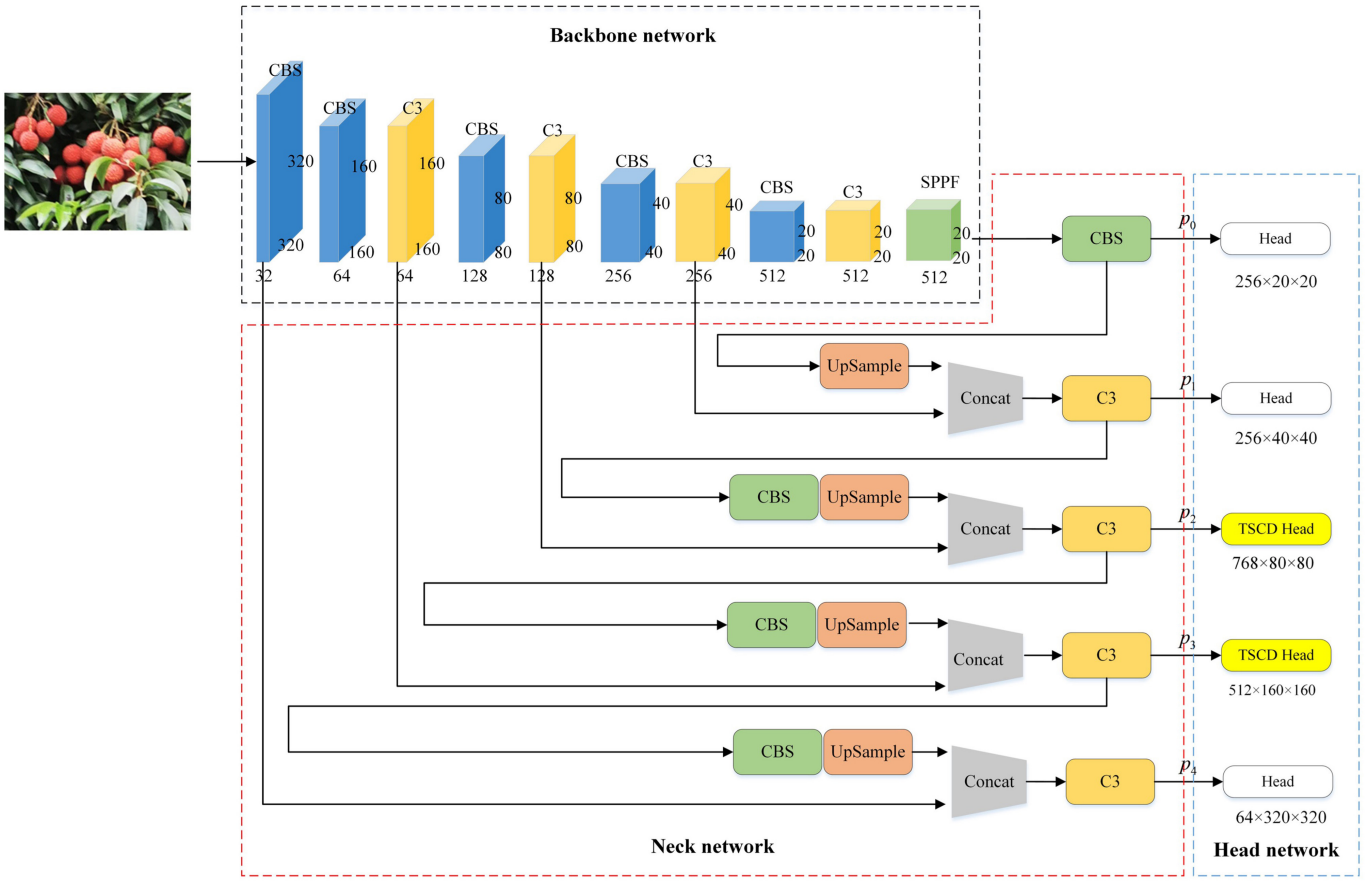
The network structure of YOLOv5-Litchi.

YOLOv5s target detection model mainly consists of Backbone network, Neck network and prediction layer. The function of the backbone network is to extract image features. The backbone network of YOLOv5s model adopts CSPDarkNet53 structure. The function of the Neck layer is to perform feature fusion on the features extracted from the backbone network. FPN (Lin et al., 2017) + PAN (Liu et al., 2018) is used to enhance the degree of feature fusion. FPN is used to transmit strong semantic features from deep to shallow, while PAN is used to transmit strong positioning features from shallow to deep, which improves the network's ability to recognize features of different feature layers. The role of the Head layer is to predict the features of three different dimensions to obtain the category and location information of the network prediction. In this paper, multi-scale features are extracted based on YOLOv5s network. Firstly, the FPN+PAN structure of Neck layer is simplified to FPN, the number of detection heads is increased from 3 to 5, and two scale TSCD detection heads of 80 × 80 (*p*_2_) and 160 × 160 (*p*_3_) are set, in order to improve the detection capability of small targets. Then, the positioning Loss and confidence Loss are optimized, and the positioning loss is replaced with EIoU Loss, and the confidence loss is replaced with Varifocal Loss (VFLoss for short), so as to improve the positioning accuracy of the detection box and further improve the ability of the network to detect dense targets. The network structure of the improved YOLOv5s network model, renamed YOLOv5-Litchi, is shown in Figure 5.

The TSCD (Two-Scale Contextual Detection) heads enhance YOLOv5-Litchi by improving small object detection and addressing dense occlusions. The TSCD structure utilizes multi-resolution feature maps generated through up-sampling and channel splicing. Specifically, the feature map of 80 × 80 resolution is combined with an up-sampled 160 × 160 map and fused with additional low-resolution data to form a rich contextual feature representation. This integration allows the TSCD heads to detect small targets more effectively by preserving spatial details and integrating multi-scale context, leading to notable improvements in precision, recall, and AP metrics. Experimental results confirm the structure's contribution to detecting challenging litchi fruit instances in natural environments.

**Figure 6:**
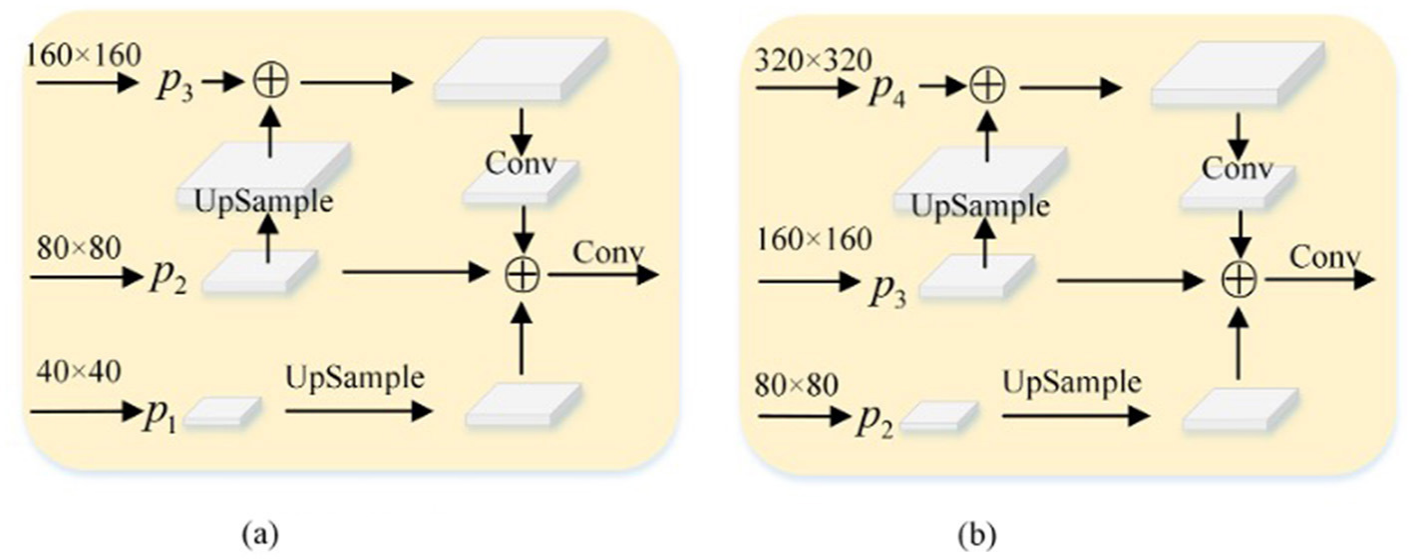
TSCD structure. **(A)** TSCD Head1 (768 × 80 × 80). **(B)** TSCD Head2 (512 × 160 × 160).

Firstly, the specific structure of TSCD Head is understood. As shown in Figure 6A, the resolution of feature figure output from the neck layer is 80 × 80. First, after up-sampling, the feature figure with a resolution of 160 × 160 is splicing in channel dimension. Then the convolution operation is used to down-sample the spliced feature map to get 256 × 80 × 80. Secondly, in order to fuse low-resolution features, feature figure with a resolution of 40 × 40 is up-sampled to get 256 × 80 × 80. Finally, the two obtained feature maps are combined with to get a 768 × 80 × 80 feature map, which is input into the Head as a new P2 feature map. The same is true for Figure 6B.

Table 2 shows the experimental comparison results of whether the Head layer uses TSCD structure. It can be seen from the table that when this structure is used in the network, AP increases by 2.4%, while the accuracy rate and recall rate increase by 1.7 and 2.1%, respectively. However, due to the addition of many up-sampling and convolution operations, the number of parameters in the model also increases accordingly.

**Table 2:** Experiments on whether to include TSCD.

**Algorithm**	**Precision**	**Recall**	**AP@0.5**	**Parameters**
Without TSCD	0.909	0.792	0.865	5,433,114
With TSCD	**0.926**	**0.815**	**0.889**	**6,388,634**

**Figure 7:**
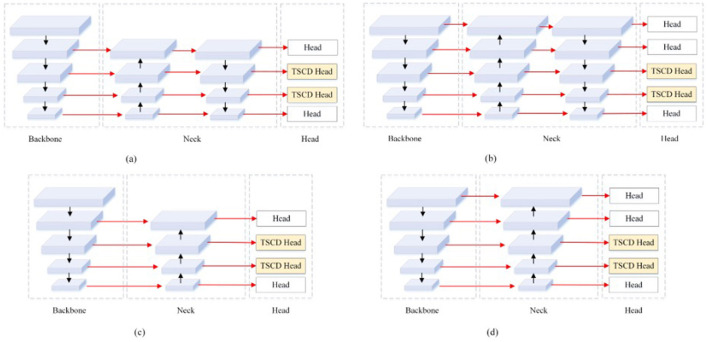
Four TSCD Head structures. **(A)** yolov5s_TSCD_V1. **(B)** yolov5s_TSCD_V2. **(C)** yolov5s_FPN_TSCD_V1. **(D)** yolov5s_FPN_TSCD_V2.

Subsequently, examine the four enhanced structures of the Neck and Head layer in YOLOv5, as depicted in Figure 7. It becomes evident that each of the four structures sets two TSCD heads as detection heads within the Head layer. Figures 7A, B illustrates that the Neck layer is the network structure of FPN+PAN, while Figures 7C, D demonstrates that the Neck layer is the network structure of FPN. Although FPN+PAN effectively integrates the features of each layer, it also introduces a substantial number of parameters. Consequently, when redesigning the Neck layer network, the approach adopted by YOLOv6 serves as a reference. YOLOv6 introduces a reduction in the three decoupling heads of classification Head(cls), regression Head(Reg), and confidence Head(obj) to two decoupling heads of classification Head(cls) and regression Head(Reg), which is equivalent to a subtraction of the network but yields superior results. In this paper, after simplifying FPN+PAN to FPN, the experiment on the litchi dataset also achieved improved results. The experimental results are presented in Table 3.

**Table 3:** TSCD head experiments.

**Model**	**Precision**	**Recall**	**AP@0.5**	**Parameters**
YOLOv5s	0.901	0.702	0.801	7,022,326
yolov5s_TSCD_V1	0.893	0.687	0.787	9,366,262
yolov5s_TSCD_V2	0.926	0.808	0.877	11,863,304
yolov5s_FPN_TSCD_V1	0.918	0.795	0.873	7,031,304
yolov5s_FPN_TSCD_V2	**0.926**	**0.815**	**0.889**	**6,388,634**

According to the experimental results, after adding one detection head and replacing two of the detection heads with TSCD detection heads, as depicted in Figure 7A, the model's performance deteriorated compared to the original YOLOv5s. However, when the FPN+PAN structure was simplified into the FPN structure, the AP, accuracy, and recall rates, respectively, increased by 7.2% in comparison to YOLOv5s. The AP value increased by 1.6%, and the accuracy and recall rates increased by 9.3%. When the number of detection heads was increased to five, the FPN structure attained optimal performance, and the AP value reached 88.9%, which was 8.8% higher than YOLOv5s.

### 3.4 Improvement of loss function

YOLOv5s will respectively classify, locate and predict the confidence of the feature map output of the Head layer, so it also corresponds to the calculation of the three losses to gradually optimize the network. However, since this paper studies single-category target detection, the loss function only includes two categories, Loss_*eiou*_ represents the positioning loss. Loss_*conf*_ is used to calculate the degree of overlap between the prediction box and the real box. Lossconf is the confidence loss, and the confidence is used to represent the reliability of the prediction box, and the prediction box with possible targets is screened. The total loss formula of YOLOv5-Litchi is as follows:


(5) $$\begin{array}{l} \text{Loss}={\text{Loss}}_{eiou}+{\text{Loss}}_{conf} \end{array}$$

Binary cross entropy loss is used for classification and confidence loss in the original YOLOv5s, and its formula is as follows:

(6) $$\begin{array}{l} \text{BCE}=\{\begin{array}{ll} -\log\left(ŷ\right) & \text{if}y=1\\ -\log\left(1-ŷ\right) & \text{if}y=0 \end{array} \end{array}$$

where *y* represents the label of the sample, 1 represents the litchi, 0 represents the background, and ŷ represents the predicted value of the network. In order to make the network adapt to the detection of dense targets, the BCE loss is replaced by the VFLoss function, the formula is as follows:

(7) $$\begin{array}{l} \text{VFL}=\{\begin{array}{ll} -q\left(q\log\left(ŷ\right)+\left(1-q\right)\log\left(1-ŷ\right)\right) & \text{if}q>0\\ -\alpha {ŷ}^{\gamma }\log\left(1-ŷ\right) & \text{if}q=0 \end{array} \end{array}$$

where ŷ is the predicted value of the network, *q* represents the label of the sample, where the γ is set to 1.5, which can be scaled by the γ factor. When YOLOv5 calculates the confidence loss, *q* is designed as the IoU between the predicted BBox and GT Box for positive samples, and *q* is designed as 0 for negative samples. It can be seen from Equation 7 that VFLoss only reduces the weight of negative samples in loss, but does not change the weight of positive samples. It makes the training pay more attention to high-quality positive samples, thus improving the detection performance.

The original YOLOv5's positioning loss adopts CIoU loss, which also takes into account the overlap area, center distance, and aspect ratio of bounding box regression. The formula is as follows:

(8) $$\begin{array}{l} L_{\text{CIoU}}=1-\text{IoU}+\frac{\rho^{2}\left(b,b^{gt}\right)}{c^{2}}+\beta \nu \end{array}$$

(9) $$\begin{array}{l} \beta =\frac{\nu }{1-\text{IoU}+\nu } \end{array}$$

(10) $$\begin{array}{l} \nu =\frac{4}{\pi^{2}}{\left(\arctan\frac{w^{gt}}{h^{gt}}-\arctan\frac{w}{h}\right)}^{2} \end{array}$$

where ρ^2^(*b, b*^*gt*^) represents the Euclidean-style distance between the center point of the prediction box and the center box, *c* represents the diagonal length containing the minimum outer box of the prediction box and the real box, β is the weight function, and ν is the aspect ratio measurement function.

The aspect ratio in CIoU uses relative values, which cannot guarantee its accuracy and does not consider the balance problem of difficult and easy samples. In order to better deal with litchi fruit detection in dense scenes, the boundary frame loss function EIoU is introduced to solve this problem. On the basis of CIoU, EIoU converts the aspect ratio into the difference between the width and height of the predicted frame and the minimum external frame. EIoU's loss function formula is as follows:


(11) $$\begin{array}{l} L_{\text{EIoU}}=L_{\text{IoU}}+L_{\text{loc}}+L_{\text{asp}}\\ \;=1-\text{IoU}+\frac{\rho^{2}\left(b,b^{gt}\right)}{c^{2}}+\frac{\rho^{2}\left(w,w^{gt}\right)}{c_{w}^{2}}+\frac{\rho^{2}\left(h,h^{gt}\right)}{c_{h}^{2}}\; \end{array}$$

**Figure 8:**
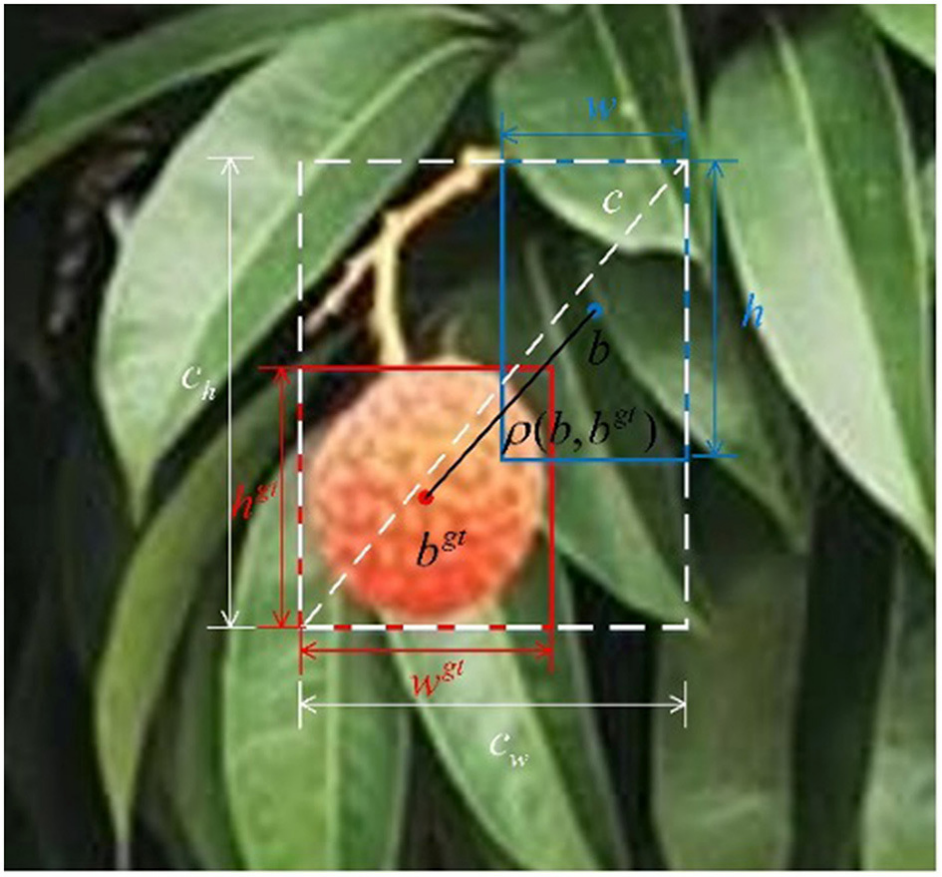
EIOU loss for bounding box regression.

Among them, $\frac{\rho^{2}\left(b,b^{gt}\right)}{c^{2}}$, $\frac{\rho^{2}\left(w,w^{gt}\right)}{c_{w}^{2}}$, and $\frac{\rho^{2}\left(h,h^{gt}\right)}{c_{h}^{2}}$ represent center point loss, width loss, and length loss, respectively. Specific parameters are shown in the Figure 8.

Generally, the process of target detection network prediction will scale the input picture to a specific size in equal proportion, for example, it can be set to the same size as the training size (640 × 640). For large-resolution pictures, if the picture is compressed in equal proportion, information will be lost, which will easily lead to the loss of small-target prediction, and the final network prediction result will be poor. And if the size of the input image is set larger, the network prediction time will also be longer.

Therefore, this paper draws on YOLT's processing method for high-resolution image prediction, and improves the model prediction. The improved prediction process is as follows: First, the input image is clipped by sliding slice, and the image is clipped into several copies in the direction of X and Y axes, each image has a certain overlap area; Then the clipped pictures are predicted separately, and each predicted result is spliced. Finally, the NMS method is used to filter out the redundant prediction boxes and get the final prediction result. This makes it possible to predict high-resolution images without loss of information by maintaining the original size and making good predictions for small targets.

**Figure 9:**
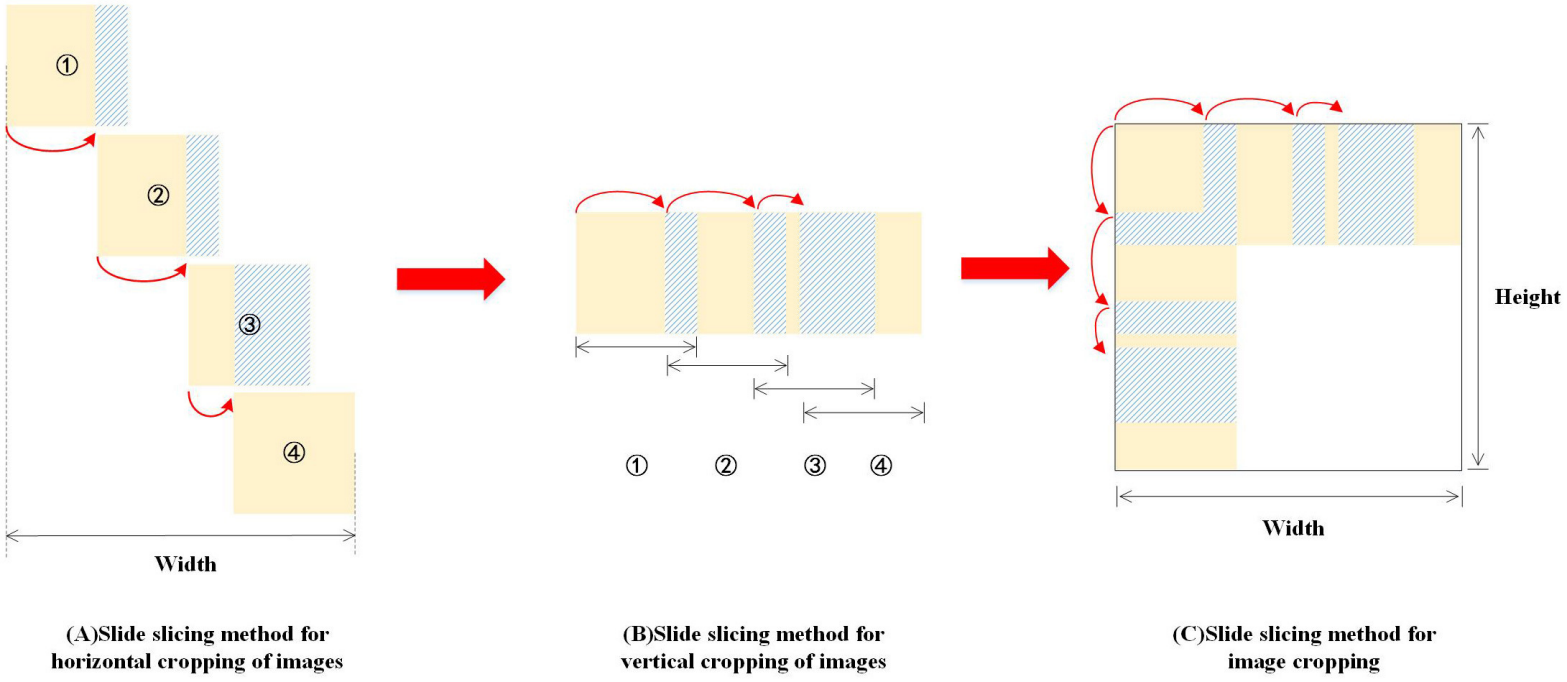
Sliding slice illustration.

The sliding slice method mainly consists of the following four steps:

**Step 1:** Define the slice size and Overlap Rate (Overlap Rate before and after the overlap rate between the two slices in proportion to the slider area);
**Step 2:** Horizontally, slices slide to the right at a certain step (Stride = 1 - Overlap Rate) (as shown in Figures 9A, B). When slices slide to the rightmost position, if the image boundary is exceeded, the Overlap Rate of slices needs to be adjusted, as shown in Figures 9A–C.
**Step 3:** In the vertical direction, similarly, slices slide vertically downward at a certain Stride = 1 - Overlap Rate. When slices exceed the image boundary, the Overlap Rate of slices is adjusted.
**Step 4:** Repeat steps 2–3 until the slice covers the entire picture.

**Figure 10:**
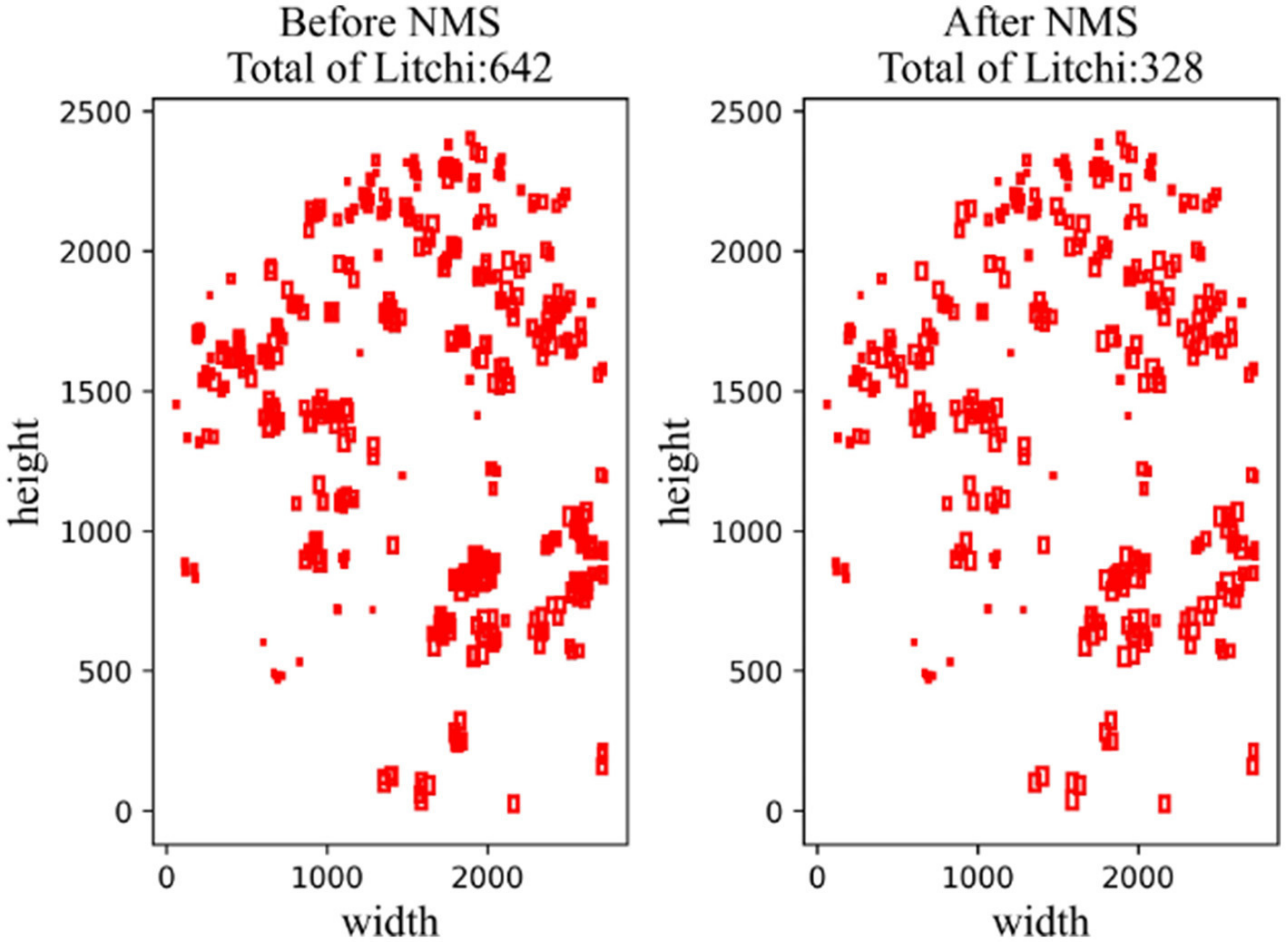
Comparison images before and after NMS.

All the images obtained by sliding slice are input into the network for prediction, and the prediction results of each image are obtained. Since each image has overlapping areas, it is necessary to use the non-maximum suppression method to screen the prediction boxes obtained, and the non-maximum suppression also has four steps:

**Step 1:** Set the threshold of the IoU.
**Step 2:** Sort all prediction boxes in the same category according to classification confidence, and select the detection box with the highest confidence at present;
**Step 3:** Traverse all other detection boxes and delete the prediction box whose IoU of the highest confidence box is higher than the threshold.
**Step 4:** Repeat steps 2–3 until all boxes are processed. As shown in Figure 10, a total of 642 litchi targets were counted after block prediction and splicing, and 328 litchi targets could be screened after NMS, among which most of the filtered prediction boxes were targets that were repeatedly predicted.

## 4 Experiments

### 4.1 Settings

In this paper, VsCode is used to build and improve the YOLOv5s network model. The processor model of the test platform is Intel Core i5-12400F, and the graphics card model is NVIDIA GTX4060. Deep learning environments such as python3.8.0, cuda11.6, and cudnn8302 have been deployed on Windows 10. Detailed device and environment parameters are shown in Table 4. All benchmarked models, including YOLOx, YOLOv6, and YOLOv8, were re-trained on the same litchi dataset to ensure a fair comparison of performance. This approach eliminates potential biases introduced by pretrained weights and ensures that the evaluation reflects the models' true capabilities on the specific task. Our used dataset was curated to ensure coverage of diverse scenarios by including images captured under varying lighting conditions, angles, and levels of occlusion, as well as different stages of litchi growth. This approach aimed to enhance the robustness of the model by representing the complexity of natural agricultural environments and addressing challenges like dense clustering and small target sizes.

**Table 4:** Hardware configuration and operating environment.

**Hardware**	**Configure**	**Environment**
System	Windows 10	Python 3.8.11
CPU	Intel(R) Core(TM) i5-12400F	PyTorch 1.12.0
GPU	RTX 4060(8G)	TorchVision 0.13.0
RAM	16G	CUDA 11.6
Hard-disk	512G	CUDNN 8302

In this paper, pre-training weights are used to improve the training speed and accuracy, and SGD is selected as the optimizer to optimize the network. The initial learning rate is set to 0.05, the image input size is set to 640 × 640, the weight decay coefficient is set to 0.0005, the batch size is set to 8, and a total of 300 epochs are iterated.

### 4.2 Evaluations

In target classification and detection tasks, Precision, Recall, AP, and F2 scores are commonly used to evaluate the generalization performance of the model. In introducing these different types of metrics, the following concepts are first introduced: True Positive (TP), False Positive (FP), True Negative (TN), and False Negative (FN), where: TP represents the true case sample and the predicted positive case sample; FP indicates that the true negative sample is incorrectly predicted to be a positive sample; TN represents the true negative sample and the predicted negative sample; FN indicates that the true case sample was incorrectly predicted as a negative case sample.

Precision represents the proportion of positive examples of correct prediction to all positive examples of prediction, which is used to measure the accuracy of the model. The calculation formula is as follows:


(12) $$\begin{array}{l} P=\frac{TP}{TP+FP} \end{array}$$

Recall refers to the proportion of correctly predicted positive samples in all actual positive samples, which is often referred to as the model's check-all rate, and its calculation formula is as follows:

(13) $$\begin{array}{l} R=\frac{TP}{TP+FN} \end{array}$$

AP (Average Precision) is a P-R curve with Recall as the horizontal axis and Precision as the vertical axis. The area under the curve is then obtained by integrating the recall rate over the interval from 0 to 1. The formula for calculating AP is as follows:


(14) $$\begin{array}{l} AP=\int_{0}^{1}P\left(R\right)dR \end{array}$$

F2 score is an indicator used to evaluate classification or detect model performance. It weights Precision and Recall. Compared with F1 score, F2 score pays more attention to model recall rate and is more suitable for litchi objects studied in this paper. The definition of F2 score is as follows:


(15) $$\begin{array}{l} F2=\frac{5PR}{4P+R} \end{array}$$

The F2 score was chosen over the F1 score in this study because it places greater emphasis on recall, which is critical in agricultural applications where minimizing missed detections is essential for accurate yield estimation. Given the dense occlusion and small target sizes in litchi fruit detection, prioritizing recall ensures a more comprehensive identification of fruits, reducing the risk of underestimating yields. The FPN+PAN structure in the Neck layer was simplified to FPN to reduce the number of parameters while maintaining effective feature fusion, and TSCD detection heads were added to improve the detection of small and occluded targets by leveraging multi-scale contextual features. Additionally, EIoU and VFLoss were introduced to replace the original loss functions, enhancing the accuracy of bounding box positioning and reducing missed detections in dense scenes.

### 4.3 Results

#### 4.3.1 Model training

**Figure 11:**
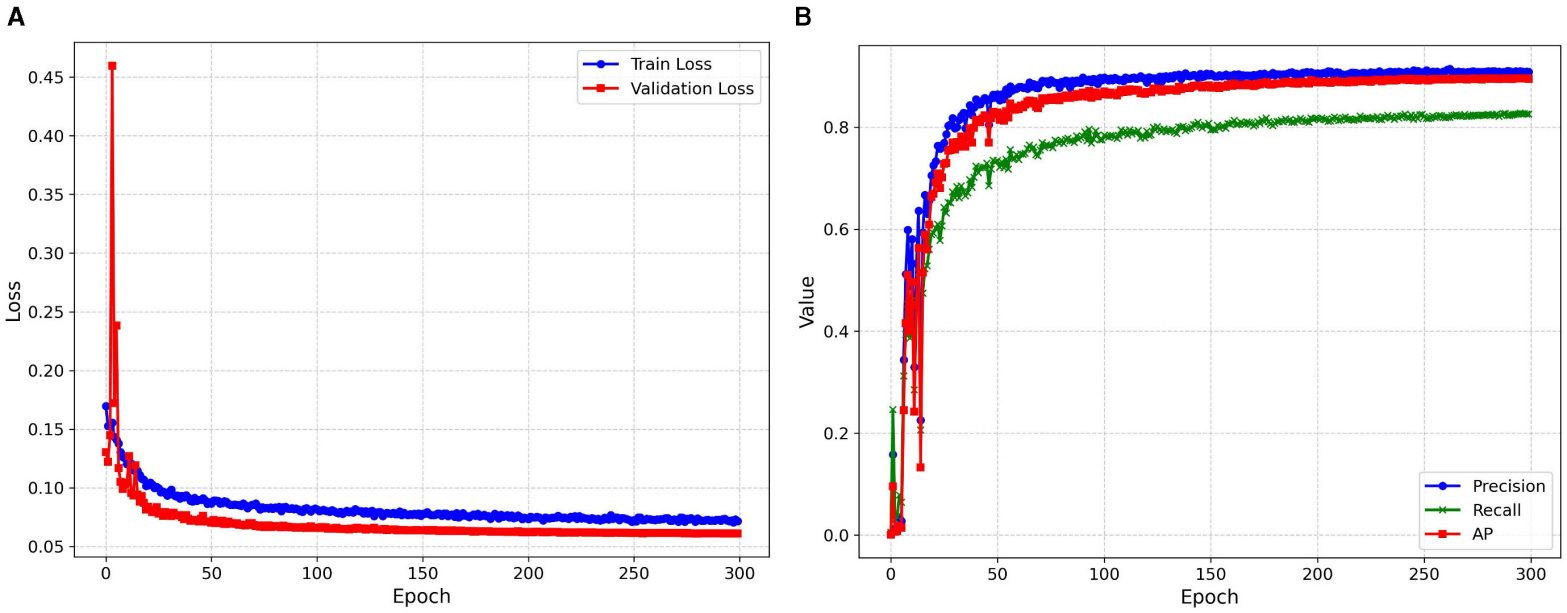
**(A)** Training and validation loss. **(B)** Trend of precision, recall, mAP0.5, and loss.

The improved YOLOv5s model proposed in this paper was used to train the data set. The curves and results of the training set loss and verification set loss functions during the training process were shown in Figure 11A. Note that the loss here represents the sum of confidence loss and positioning loss. The change curves of verification set accuracy rate, recall rate and AP during the training process are shown in Figure 11B.

As can be seen from Figure 11A, the loss oscillation of the first 50 epoch verification sets is more severe than that of the training set, but the loss values all show a downward trend. When the number of iterations reaches 200, the model loss values no longer decrease significantly, and all evaluation indexes also tend to be stable.

Figure 11B shows that the accuracy rate, recall rate, and AP of the first 50 epochs show a rapid upward trend. When the number of iterations reaches 150, the model gradually converges, and the final AP is 89.6%, the accuracy rate is 91.0%, and the recall rate is 82.5%.

#### 4.3.2 Ablation study

We design four groups of ablation experiments with different models, and the experimental results were shown in Table 5.

**Table 5:** Result of the ablation experiments.

**Nums**	**Model**	**Precision**	**Recall**	**F2**	**AP@0.5**
1	YOLOv5s	0.901	0.702	0.734	0.801
2	1+FPN+SmallObj	0.909	0.792	0.813	0.865
3	2+TSCD Head	0.926	0.815	0.835	0.889
4	3+VFLoss+EIoU	0.910	0.825	0.841	0.896

As can be seen from Table 5, the FPN+PAN of Neck layer in YOLOv5s was simplified into FPN structure and expanded into five detection heads of different scales. Compared with YOLOv5s, AP value increased by 6.4% and recall rate increased by 9.0%. This also shows that the addition of small target detection layer can effectively reduce the missed rate of the network, especially improve the detection accuracy of small targets. The introduction of TSCD detection head can further improve the accuracy of the network, and the accuracy rate, recall rate and AP increase by 1.7, 2.3, and 2.4%, respectively, indicating that the TSCD structure has the ability to fully integrate the context feature information, and this step of improvement is positive and effective. The final improvement is mainly for the loss function of the network, and the main contribution is reflected in the improvement of the recall rate. Even if the accuracy rate is reduced, this step of improvement can reduce the problem of missing detection and less detection, which is suitable for the improvement of intensive scenes and small target direction.

#### 4.3.3 Comparison of different detection algorithms

In order to compare the improved model with different algorithm models, analyze the performance of different algorithms and explore the superiority of the improved algorithm in this study, the current mainstream target detection algorithms, including YOLOX, YOLOv6, YOLOv8, and YOLOv5s, are selected for test comparison, and the results are shown in Table 6. As can be seen from the table, the average accuracy of YOLOv5s-litchi model is 9.5, 4, 6.3, and 3.7 percentage points higher than that of other models, respectively. Among them, the accuracy rates of all models are close, with the lowest being 88%, while the recall rates differ greatly. Thus, the difficulty of this data set lies in dense and obscured targets. The YOLO-Litchi model increases the recall rate from 70.2 to 82.5%, which also shows that the improved method in this paper has certain effect.

**Table 6:** Performance comparison of the state-of-the-art models.

**Model**	**Precision**	**Recall**	**AP@0.5**	**Model size**
YOLOv5	0.901	0.702	0.801	14.4MB
YOLOX	0.897	0.782	0.856	16.3MB
YOLOv6	0.883	0.741	0.833	32.8MB
YOLOv8	0.88	0.776	0.859	22.5MB
YOLO-litchi	0.910	0.825	0.896	15.7MB

**Figure 12:**
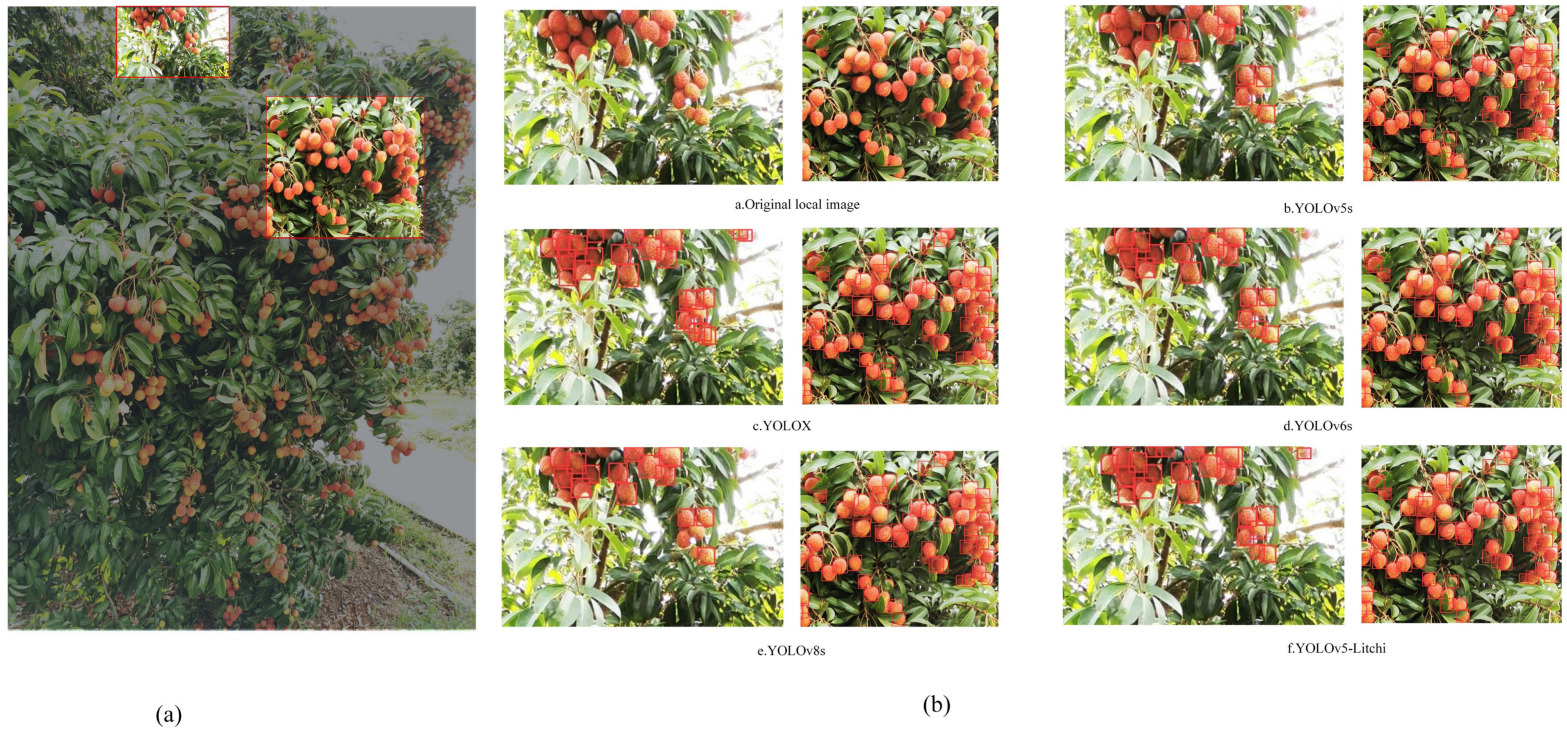
Recognition effects of different models on litchi. **(A)** Original image. **(B)** Detection effect.

Figure 12A shows the original image, which is used for prediction, and Figure 12B shows two local images which are extracted from the predicted image for analysis. According to the predicted results, we can see: The model presented in this paper has a good comprehensive detection performance. As litchi fruit in the first figure takes up fewer pixels in the original figure and the target is also relatively small, YOLOX model can detect most of them but has the problem of repeated detection, and other models basically cannot detect them. However, this model has a relatively large advantage in detecting small targets, and there is no missing detection. It is shown that increasing the small target scale and using TSCD structure optimization prediction head are helpful for small target detection. The second figure is mainly about the detection effect of litchi under dense scenes. Compared with other models, the optimized model has less missed detection, and litchi with occlusion can also be detected. Secondly, compared with other models, the detected litchi prediction frame is more suitable for litchi, indicating that the improved loss function is helpful to the detection of litchi fruit.

#### 4.3.4 Sliding slices experiment

**Figure 13:**
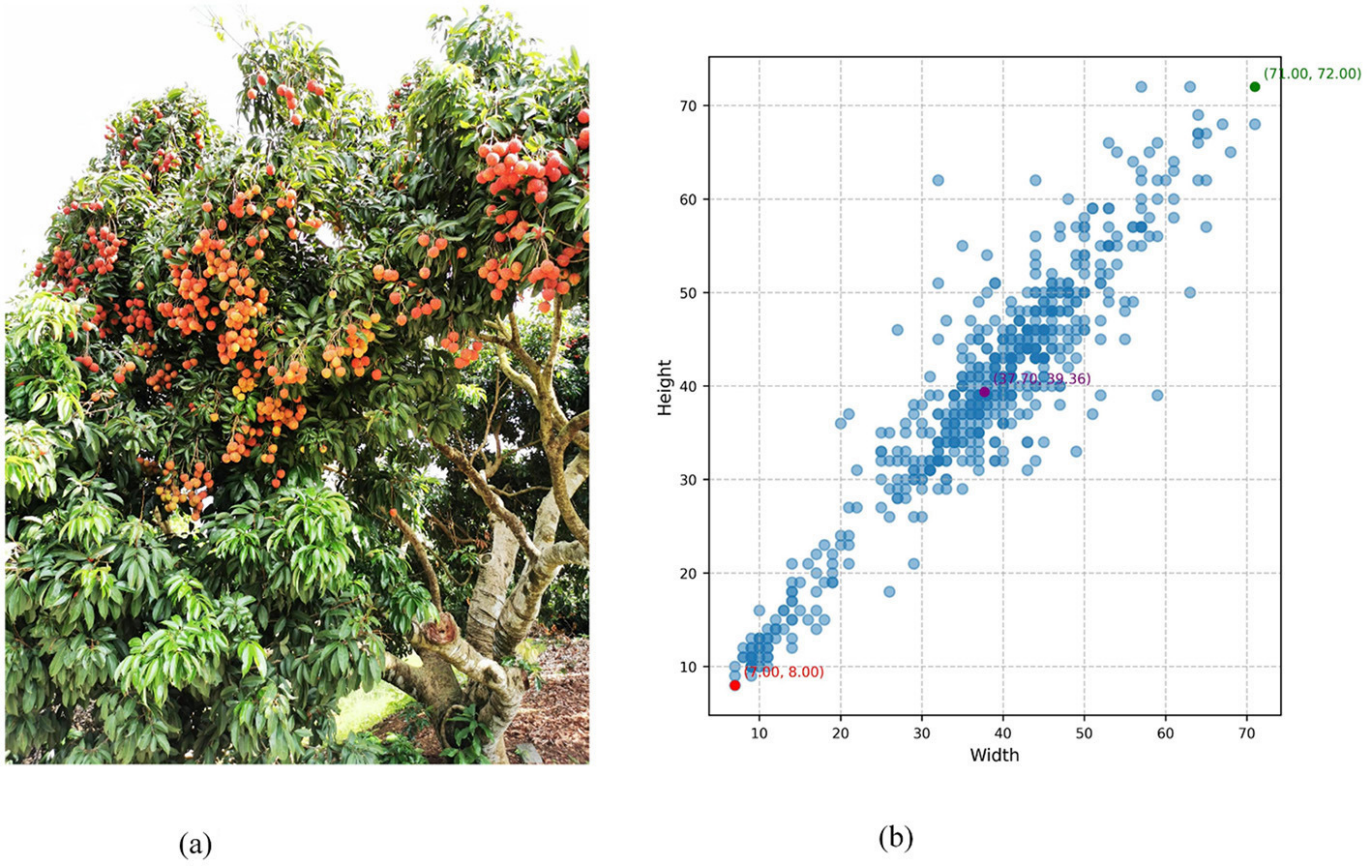
Sliding slice predictions with original image and label information. **(A)** Original images. **(B)** Label distribution.

In order to facilitate the comparison between the predicted results and actual labels, Figure 13 shows a data specially relabeled for small targets, so small litchi targets will also be labeled. It can be seen from the following figure that there are a total of 571 litchi targets, the maximum width and height of which is 71 × 72 pixels, the average pixel is 37.7 × 39.36, and the pixel of the original image is 2,736 × 3,648.

**Figure 14:**
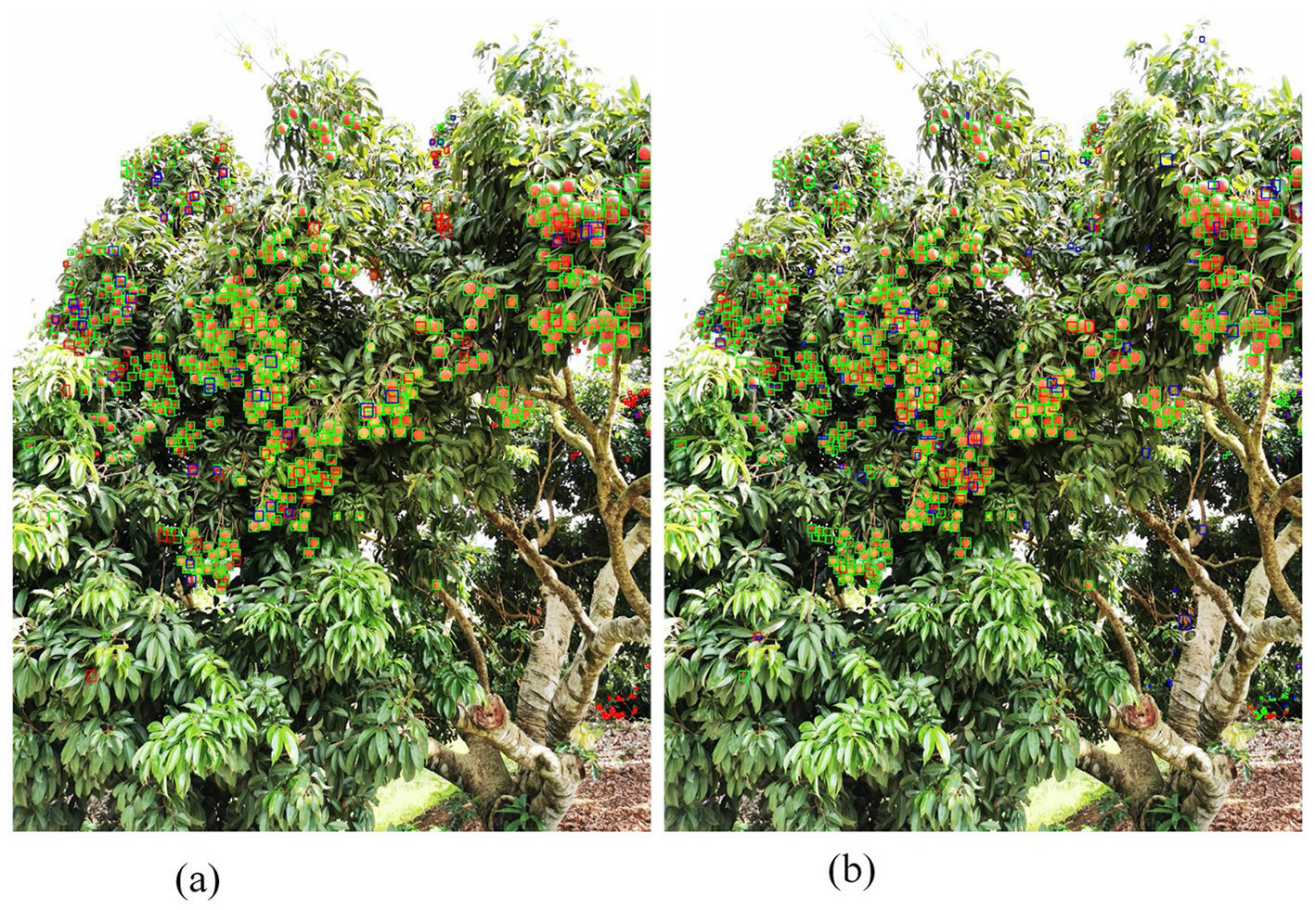
Comparison chart of sliding slice predictions and direct predictions. **(A)** YOLOv5-Litchi. **(B)** Slicing detects.

Figure 14 shows two different reasoning methods. One is to directly scale the image to 640 × 640 pixels for reasoning, and the predicted result is as shown in Figure 14A; the other is to reason by slicing the slider, setting the size of the slider to 768 and the overlap rate to 0.3. The predicted results are shown in Figure 14B. The red box indicates a missed target, the blue box indicates a misdetected target (the IoU of the prediction box and label is less than the threshold), and the green box indicates a positive target.

When the IoU threshold is set to 0.55, the statistical prediction of the two methods is shown in Table 7: (1) The direct reasoning method has 432 positive checks, 139 missed checks, and 41 false checks; (2) The sliding block method has 496 positive tests, 75 missed tests, and 88 false tests; (3) Experiments show that the sliding block method can effectively reduce missed detection and improve positive detection at the same time, but it will also bring some false detection. The reasons are analyzed. On the one hand, the model will misjudge due to the cutting of the target caused by the sliding block slicing method, and on the other hand, the model will misdetect large targets because the data set is not perfect.

**Table 7:** Comparison of prediction results between sliding slice and direct prediction.

**Prediction method**	**TP (right)**	**FP (error)**	**FN (missing)**
YOLOv5-Litchi	432	41	139
YOLOv5-Litchi+sliding detects	496	88	75

It can be conclude that, the practical significance of the proposed model lies in its ability to detect small and occluded litchi fruits with high accuracy, which is crucial for reliable yield estimation and effective resource allocation in agricultural practices. By addressing challenges in dense and complex natural environments, the model provides a robust solution for automating fruit detection, ultimately supporting improved decision-making in crop management and harvest planning.

## 5 Conclusion

In this paper, an enhanced litchi fruit detection model, YOLOv5-Litchi, was developed upon the foundation of YOLOv5s. The Neck layer was simplified from the FPN+PAN structure to the FPN structure. Additionally, feature fusion was further strengthened by incorporating a small target detection Head and replacing the TSCD Head in the head layer. Finally, the EIoU Loss and confidence loss of YOLOv5 were replaced by VFLoss for positioning loss of YOLOV5. Furthermore, the sliding slice method was employed experimentally to predict images. Through an ablation test of the improved model and a comparison with other target detection models, the following conclusions can be drawn: (1) The average accuracy of the YOLOv5-Litchi algorithm model is 89.6%, with an accuracy rate of 91.0% and a recall rate of 82.5%. Compared to the original model YOLOv5s, the mean average precision (mAP), accuracy rate, and recall rate are respectively increased by 9.5, 0.9, and 12.3 percentage points. In comparison with other algorithms, the improved network exhibits distinct improvements, primarily focusing on enhancing the recall rate and AP value of the network, thereby reducing the missed detection rate. (2) In terms of practical detection performance, the improved network exhibits a reduced number of missed targets and a more accurate prediction frame, indicating its suitability for litchi fruit detection. Furthermore, the experimental results of the sliding slice method demonstrate that the sliding block clipping and splicing method can effectively enhance the ability of small target detection.

## Data Availability

The original contributions presented in the study are included in the article/supplementary material, further inquiries can be directed to the corresponding author.
